# Skill Learning by Autonomous Robotic Playing Using Active Learning and Exploratory Behavior Composition

**DOI:** 10.3389/frobt.2020.00042

**Published:** 2020-04-03

**Authors:** Simon Hangl, Vedran Dunjko, Hans J. Briegel, Justus Piater

**Affiliations:** ^1^Intelligent and Interactive Systems, Department of Informatics, University of Innsbruck, Innsbruck, Austria; ^2^LIACS, Leiden University, Leiden, Netherlands; ^3^Institute for Theoretical Physics, University of Innsbruck, Innsbruck, Austria

**Keywords:** active learning, hierarchical models, skill learning, reinforcement learning, autonomous robotics, robotic manipulation, behavior composition

## Abstract

We consider the problem of autonomous acquisition of manipulation skills where problem-solving strategies are initially available only for a narrow range of situations. We propose to extend the range of solvable situations by autonomous play with the object. By applying previously-trained skills and behaviors, the robot learns how to prepare situations for which a successful strategy is already known. The information gathered during autonomous play is additionally used to train an environment model. This model is exploited for active learning and the generation of novel preparatory behaviors compositions. We apply our approach to a wide range of different manipulation tasks, e.g., book grasping, grasping of objects of different sizes by selecting different grasping strategies, placement on shelves, and tower disassembly. We show that the composite behavior generation mechanism enables the robot to solve previously-unsolvable tasks, e.g., tower disassembly. We use success statistics gained during real-world experiments to simulate the convergence behavior of our system. Simulation experiments show that the learning speed can be improved by around 30% by using active learning.

## 1. Introduction

Humans perform complex object manipulations so effortlessly that at first sight it is hard to believe that this problem is still unsolved in modern robotics. This becomes less surprising if one considers how many different abilities are involved in human object manipulation. These abilities span from control (e.g., moving arms and fingers, balancing the body), via perception (e.g., vision, haptic feedback) to planning of complex tasks. Most of these are not yet solved in research by themselves, not to speak of combining them in order to design systems that can stand up to a comparison with humans. However, there is research by Meeussen et al. (2010), Mülling et al. (2013), Abu-Dakka et al. (2014), and Hangl et al. (2014, 2015) on efficiently solving specific problems (or specific classes of problems).

Not only the performance of humans is outstanding—most manipulation skills are learned with a high degree of autonomy. Humans are able to use experience and apply the previously learnt lessons to new manipulation problems. In order to take a step toward human-like robots we introduce a novel approach for autonomous learning that makes it easy to embed state-of-the-art research on specific manipulation problems. Further we aim to combine these methods in a unified framework that is able to solve increasingly complex tasks.

In this work we are inspired by the behavior of infants at an age between 8 and 12 months. Piaget (1952) identified different phases of infant development. A phase of special interest is the *coordination of secondary schemata* which he identifies as the stage of first actually intelligent behavior. At this stage infants combine skills that were learned earlier in order to achieve more complex tasks, e.g., kicking an obstacle out of the way such that an object can be grasped. In this stage children do not predict the outcome of actions and check the corresponding pre- and post-conditions (Piaget, 1952) as it is done in many planning systems (e.g., by Fainekos et al., 2005; Ferrein and Lakemeyer, 2008; Kress-Gazit et al., 2009). To them it is only important to know that a certain combination of manipulations is sufficient to achieve a desired task. The environment is prepared such that the actual skill can be applied without a great need for generalization. Even adults exhibit a similar behavior, e.g., in sports. A golf or tennis player will always try to perform the swing from similar positions relative to the ball. She will position herself accordingly instead of generalizing the swing from the current position. This is equivalent to concatenating two behaviors, walking toward the ball and executing the swing.

In previous work by Hangl et al. (2016) we introduced an approach that is loosely inspired by this paradigm. The robot holds a set of *sensing behaviors, preparatory behaviors*, and *basic behaviors*, i.e., behaviors that solve a certain task in a narrow range of situations. It uses the sensing behaviors to determine the state of the environment. Depending on the state, a preparatory behavior is used to bring the environment into a state in which the task can be fulfilled by simple replay of the basic behavior. The robot does not need to learn how to generalize a basic behavior to every possibly observable situation. Instead, the best combination of sensing behaviors and preparatory behaviors is learned by autonomous play.

We phrase playing as a reinforcement learning (RL) problem, in which each rollout consists of the execution of a sensing behavior, a preparatory behavior and the desired basic behavior. Each rollout is time consuming, but not necessarily useful. If the robot already knows what to do in a specific situation, performing another rollout in this situation does not help to improve the policy. However, if another situation is more interesting, it can try to prepare it and continue the play, i.e., *active learning*. Our original approach is model-free, which makes it impossible to exhibit such a behavior. In this paper we propose to learn a forward model of the environment which allows the robot to perform transitions from *boring* situations to *interesting* ones. We use the terms *boring*/*interesting* as metaphors for situations in which the robot achieves already high/still low success rates and therefore there is not much/much left to learn. These terms do not attribute emotional responses to the agent.

Our work strongly relates to work in active learning in robotics (c.f. Sutton, 1990; Kaelbling, 1993; Koenig and Simmons, 1993; Schaal and Atkeson, 1994), where the robot aims to improve its environment model by asking for or creating situations the maximize the learning rate. However, there is an important distinction: the work presented in this paper is not centered on creating an environment model as the core is model-free. In our approach, active learning capabilities are only unlocked if the model-free core is confident it can resolve the situation, i.e., the robot is bored, and the environment model is mature enough so the robot is able to plan a transition to an interesting state. The robots major goal is not to learn a model of the environment but to solve the task at hand. It will only exploit the model if the model-free approach does not provide a sufficient success rate before the environment model becomes mature. In this case plans can be created to switch between states as required.

Another issue is the strict sequence of phases: *sensing* → *preparation* → *basic behavior*. In this work we weaken this restriction by enabling the robot to generate novel preparatory behaviors composed of other already known behaviors. The environment model is used to generate composite behaviors that are potentially useful instead of randomly combining behaviors.

We illustrate the previously described concepts with the example of book grasping. This task is hard to generalize but easy to solve with a simple basic behavior in a specific situation. The robot cannot easily get its fingers underneath the book in order to grasp it. In a specific pose, the robot can squeeze the book between two hands, lifting it at the spine and finally slide its fingers below the slightly-lifted book. Different orientations of the book would require adaption of the trajectory. The robot would have to develop some understanding of the physical properties, e.g., that the pressure has to be applied on the spine and that the direction of the force vector has to point toward the supporting hand. Learning this degree of understanding from scratch is a very hard problem.

Instead, we propose to use preparatory behaviors, e.g., *rotating the book by 0*°*, 90*°*, 180*°*, or 270*°, in order to move it to the correct orientation (ϕ = 0°) before the basic behavior is executed. The choice of the preparatory behavior depends on the book's orientation, e.g., ϕ ∈ {0°, 90°, 180°, 270°}. The orientation can be estimated by sliding along the book's surface, but not by poking on top of the book. The robot plays with the object and tries different combinations of sensing behaviors and preparatory behaviors. It receives a reward after executing the basic behavior and continues playing. After training, the book grasping skill can be used as preparatory behavior for other skills in order to build hierarchies.

If the robot already knows well that it has to perform the behavior *rotate 90*° if ϕ = 270° and is confronted with this situation again, it cannot learn anything any more, i.e., it is *bored*. It can try to prepare a more interesting state, e.g., ϕ = 90° by executing the behavior *rotate 180*°. We refer to active exploration of more interesting states as *active learning*. Further, if only the behavior *rotate 90*° is available, the robot cannot solve the situations ϕ ∈ {90°, 180°} by executing a single behavior. However, it can use behavior compositions in order to generate the behaviors *rotate 180*° and *rotate 270*°.

## 2. Related Work

### 2.1. Skill Chaining and Hierarchical Reinforcement Learning

Sutton et al. (1999) introduced the *options* framework for skill learning in a RL setting. Options are actions of arbitrary complexity, e.g., atomic actions or high-level actions, such as grasping, modeled by semi-Markov decision processes (SMDP). They consist of an option policy, an initiation set indicating the states in which the policy can be executed, and a termination condition that defines the probability of the option terminating in a given state. Options are orchestrated by Markov decision processes (MDP), which can be used for planning to achieve a desired goal. This is related to our notion of behaviors, however, behaviors are defined in a loser way. Behaviors do not have an initiation set and an explicit termination condition. Behaviors are combined by grounding them on actual executions by playing instead of concatenating them based on planning. Konidaris and Barto (2009) embedded so called *skill chaining* into the options framework. Similar to our work, options are used to bring the environment to a state in which follow-up options can be used to achieve the task. This is done by standard RL techniques, such as Sarsa and Q-learning. The used options themselves are autonomously generated, however, as opposed to our method, the state space is pre-given and shared by all options. Lee et al. (2013) developed a framework for imitation learning in which the taught actions are mapped to probabilistic symbols. Skill chaining is achieved by combining these symbols with probabilistic activity grammars. The robot searches for frequently common sub-patterns, splits the actions and generates new symbols. In contrast, in our approach the skills are shown per task and behaviors are not split but combined when needed. Instead of autonomously creating novel options, Konidaris et al. (2011) extended this approach by deriving options from segmenting trajectories trained by demonstration. On a more abstract level, Colin et al. (2016) investigated creativity for problem-solving in artificial agents in the context of hierarchical reinforcement learning by emphasizing parallels to psychology. They argue that hierarchical composition of behaviors allows an agent to handle large search spaces in order to exhibit creative behavior.

### 2.2. Model-Free and Model-Based Reinforcement Learning in Robotics

Our work combines a model-free playing system and a model-based behavior generation system. Work on switching between model-free and model-based controllers was proposed in many areas of robotics (e.g., by Daw et al., 2005; Dollé et al., 2010; Keramati et al., 2011; Caluwaerts et al., 2012a,b; Renaudo et al., 2014, 2015). The selection of different controllers is typically done by measuring the uncertainty of the controller's predictions. Renaudo et al. (2014, 2015) proposed switching between so called model-based and model-free experts, where the model is learned over time. The switching is done randomly, or by either majority vote, rank vote, Boltzmann Multiplication or Boltzmann Addition. Similar work has been done in a navigation task by Caluwaerts et al. (2012a,b). Their biologically inspired approach uses three different experts, namely a taxon expert (model-free), a planning expert (model-based), and an exploration expert, i.e., exploring by random actions. A so called *gating network* selects the best expert in a given situation. All these methods hand over the complete control either to a model-based or a model-free expert. In contrast, our method always leaves the control with the model-free playing system which makes the final decision on which behaviors should be executed. The model-based system, i.e., behavior generation using the environment model, is used to add more behaviors for model-free playing. This way, the playing paradigm can still be maintained while enabling the robot to come up with more complex proposals in case the task cannot be solved by the model-free system alone.

Dezfouli and Balleine (2012) sequence actions and group successful sequences to so-called *habits*. Roughly speaking, task solutions are generated by a dominant model-based RL system and are transformed to atomic habits if they were rewarded many times together. In contrast, the main driving component of our method is a model-free RL system which is augmented with behavioral sequences by a model-based system. This way, the robot can deal with problems without requiring an environment model while still being able to benefit from it.

### 2.3. Developmental Robotics

Our method shares properties with approaches in developmental robotics. A common element is the concept of *lifelong learning*, in which the robot develops more and more complex skills by interacting with the environment autonomously. Wörgötter et al. (2015) proposed the concept of structural bootstrapping in which knowledge acquired in earlier stages of the robot's life is used to speed up future learning. Weng (2004) provides a general description of a *self-aware and self-affecting agent* (SASE). He describes an agent with *internal* and *external* sensors and actuators, respectively. It is argued that autonomous developmental robots need to be SASE agents and concrete implementations are given, e.g., navigation or speech learning. Our concept of boredom is an example of a paradigm, in which the robot decides on how to proceed based on *internal sensing*. Ivaldi et al. (2014) developed an architecture for learning object properties and models through life-long learning and intrinsic motivation based on physical interaction with the environment. Moulin-Frier et al. (2017) developed a cognitive architecture to solve the symbol ground problem. They defined a layered architecture composed of structural models that handle functional tasks. Similar to our approach, their architecture allows the use of state-of-the-art models. Their approach acts on a higher cognitive level but requires definition of the structural models while in our approach arbitrary structural modules can be used as long as they are hidden in behaviors.

In general, developmental robotics shares some key concepts with our method, e.g., lifelong learning, incremental development or internal sensing. For a detailed discussion we refer to a survey by Lungarella et al. (2003).

### 2.4. Active Learning in Robotics

In active learning the agent can execute actions which have an impact on the generation of training data (c.f. Thrun, 1995). In the simplest case, the agent explores the percept-action space by random actions (Whitehead, 2014). The two major active learning paradigms, i.e., query-based and exploration-based active learning, differ in the action selection mechanism. Query-based learning systems request samples, e.g., by asking a supervisor for it. Typically, the request is based on the agent's uncertainty (Atlas et al., 1990; Cohn, 1994; Cohn et al., 1996). Chao et al. (2010) adopt query-based active learning for *socially guided machine learning* in robotics. Task models are trained by interaction with a human teacher, e.g., classifying symbols assigned to tangram compounds. The robot could prepare a desired sample by itself, i.e., arranging interesting tangram compounds and asking the teacher for the class label. In contrast to our method, this is not done in practice, but the robot describes the desired compound.

Exploration-based active learning paradigms, on the other hand, select actions in order to reach states with maximum uncertainty (Sutton, 1990; Kaelbling, 1993; Koenig and Simmons, 1993; Schaal and Atkeson, 1994). Salganicoff et al. (1996) and Morales et al. (2004) used active learning for grasping. It was used to learn a prediction model of how good certain grasp types will work in a given situation. All these works deal with how to select actions such that a model of the environment can be trained more effectively. In our approach the training of the environment model is not the major priority. It is a byproduct of the autonomous play and is used to speed up learning and generate behaviors on top of the playing system.

Kroemer et al. (2010) suggested a hybrid approach of active learning and reactive control for robotic grasping. Active learning is used to explore interesting poses using an *upper confidence bound* (UCB) (Sutton and Barto, 1998; Auer et al., 2002) policy that maximizes the *merit*, i.e., the sum of the expected reward mean and variance. The actual grasps are executed by a reactive controller based on *dynamic movement primitives* (DMPs) (c.f. Schaal, 2006), using attractor fields to move the hand toward the object and detractor fields for obstacle avoidance. This approach is tailored to a grasping task, in which the autonomous identification of possible successful grasps is hard due to high-dimensional search spaces. In contrast, our approach is acting on a more abstract level in which the described grasping method can be used as one of the preparatory behaviors. A more detailed investigation of active learning is outside the scope of this paper and can be found in a survey by Settles (2010). Special credit shall be given to work on *intrinsic motivation* by Barto et al. (2004), Stoytchev and Arkin (2004), Oudeyer et al. (2007), Schembri et al. (2007), Baranes and Oudeyer (2009), Lopes and Oudeyer (2010), Ivaldi et al. (2014), Ribes et al. (2015), and Ugur and Piater (2016). It is a flavor of active learning which is commonly applied in autonomous robotics. Instead of maximizing the uncertainty, these methods try to optimize for intermediate uncertainty. The idea is to keep the explored situations simple enough to be able to learn something, but complex enough to observe novel properties. Schmidhuber (2010) provides a sophisticated summary of work on intrinsic motivation and embeds the idea into a general framework. He states that many of these works optimize some sort of intrinsic reward, which is related to the improvement of the prediction performance of the model. This is closely related to our notion of boredom, in which the robot rejects the execution of skills in a well-known situation for the sake of improving the policy in other situations. He further argues that such a general framework can explain concepts like creativity and fun.

*Self-organization* (Der and Martius, 2006; Martius et al., 2007, 2013; Martius and Herrmann, 2012) is a concept contrasting active learning and intrinsic motivation. This paradigm is not based on random exploration but on deterministic policies for explorative action selection in order to overcome the curse of dimensionality. For example this was done by Bialek et al. (2001) by optimizing a generalization the of predictive information measure (PI), or by Martius et al. (2013) by using neural networks for modeling the system dynamics and gradient descent.

### 2.5. Planning

Many of the previously mentioned methods are concerned with training forward models, which in consequence are used for planning in order to achieve certain tasks. Ugur and Piater (2015) proposed a system that first learns action effects from interaction with the objects and is trained to predict single-object cagetories from visual perception. In a second stage, multi-object interaction effects are learned by using the single-object categories, e.g., two solid objects can be stacked on top of each other. Discrete effects and categories are transformed into a PDDL description. Symbolic planning is used to create complex manipulation plans, e.g., for creating high towers by stacking. Konidaris et al. (2014) suggest a method in which symbolic state representations are completely determined by the agent's environment and actions. They define a symbol algebra on the states derived from executed actions that can be used for high-level planning in order to reach a desired goal. Konidaris et al. (2015) extend this set-based formulation to a probabilistic representation in order to deal with the uncertainty observed in real-world settings. A similar idea is present in our model-free approach, where the selection of sensing behaviors and the semantics of the estimated states depends on the desired skill.

All these approaches provide a method to build a bridge from messy sensor data and actions to high-level planning systems for artificial intelligence. In order do to so, similar to our approach, abstract symbols are used. However, these systems require quite powerful machinery in order to provide the required definition of pre- and post-conditions for planning. In our approach the robot learns a task policy directly, which is augmented by a simple planning-based method for composite behavior generation.

## 3. Problem Statement

The goal is to increase the scope of situations in which a *skill* can be applied by exploiting *behaviors*. A behavior *b* ∈ *B* maps the complete (and partially unknown) state of system **e** ∈ *A* × *E* to another state **e**′ ∈ *A* × *E* with

(1) $$\begin{array}{l} b:A\times E\mapsto A\times E \end{array}$$

The sets *A, E* denote the internal state of the robot and the external state of the environment (e.g., present objects), respectively. We aim for autonomous training of a goal-directed behavior, i.e., a *skill*. This requires a notion of success, i.e., a success predicate. We define a skill σ = (*b*^σ^, Success^σ^) as a pair of a *basic behavior* *b*^σ^, i.e., a behavior that solves the task in a narrow range situations, and a predicate

(2) $$\begin{array}{l} {\text{Success}}^{\sigma }\left(b^{\sigma }\left(\text{e}\right)\right)=true \end{array}$$

with **e** ∈ *D*^σ^. The non-empty set *D*^σ^ ⊆ *A* × *E* is the set of all states in which the skill can be applied successfully, i.e., all states in which the fixed success predicate holds. We call the set *D*^σ^ the *domain of applicability* of the skill σ. The goal is to *extend the domain of applicability* by finding behavior compositions *b*_*l*_ ∘⋯∘ *b*_2_∘ *b*_1_ with the property

(3) $$\begin{array}{l} {\text{Success}}^{\sigma }\left(b_{l}\circ \cdots \circ b_{2}\circ b_{1}\circ b^{\sigma }\left(\text{e}\right)\right)=true \end{array}$$

with *b*_*i*_ ∈ *B* and **e** ∈ *D*^′σ^ ⊆ *A* × *E* such that *D*^′σ^ ⊋ *D*^σ^, i.e., the domain of applicability is *larger* than before. A behavior composition $b_{l}\circ \cdots \circ b_{2}\circ b_{1}\circ b^{\sigma }$ is a behavior itself and therefore can be used to extend the domain of applicability of other skills. This way, skills can become more and more complex over time by constructing skill hierarchies.

## 4. Contribution

We extend an approach for skill learning by autonomous playing introduced by Hangl et al. (2016). It uses only one preparatory behavior per state, i.e., allowing only behavior compositions of length *l* = 1 (c.f. Equation 3). This limitation enables the robot to perform model-free exploration due to the reduced search space. Allowing behavior compositions of length *l* > 1 causes the learning problem to be intractable, but would help to solve more complex tasks.

Approaches dealing with problems of this complexity have to strongly reduce the search space, e.g., by symbolic planning by Ugur and Piater (2015) and Konidaris et al. (2014, 2015). We do not follow a planning-based paradigm in the traditional sense. The playing-based exploration of actions remains the core component of the system. In order to allow behavior compositions of length *l* > 1 while still keeping the advantage of a small search space, we introduce a separate model-based system which generates potentially useful behavior compositions. A forward model of the environment is trained with information acquired during autonomous play. The environment model is used to generate new behavior compositions that might be worth trying out. The ultimate decision whether a behavior composition is used, however, is still up to the playing-based system. This way, the advantages of model-free and model-based approaches can be combined:

1. Behavior compositions of arbitrary length can be explored without having to deal with the combinatorial explosion of possible behavior compositions.
2. No or only weak modeling of the environment is required because the playing-based approach alone is still stable and fully-functional.
3. Exploration beyond the modeled percept-action space can still be done, e.g., a book flipping action can be used to open a box (Hangl et al., 2016).

Proposals for novel preparatory behaviors are considered proportional to their expected usefulness. This enables the robot to first consider more conservative plans and to explore more unorthodox proposals in later stages. We refer to this procedure as *compositional generation of behavior proposals*. We relate to a principal investigation of creative machines by Briegel (2012), in which robots use a memory to propose combinations of previous experiences in order to exhibit *new* behavioral patterns.

We further exploit the environment model for speeding up the learning process by *active learning*. The robot can be *bored* of certain situations and is not only asking for different situations but also prepares them by itself. Whether or not the robot is bored is part of the internal state **e**_*A*_ ∈ *A* of the robot, which is made explicit in Equation (1).

We believe that a lifelong learning robot must go through different developmental stages of increasing complexity. Optimally, these stages are not hard-coded to the system but emerge automatically over the course of the robot's life. We extend our original system such that these additional mechanisms are exploited as soon as the robot is ready for it, i.e., the environment model is mature enough.

## 5. Preliminaries

For better understanding of the remainder of the paper, we introduce the concept of perceptual states. We further provide a brief description of the core reinforcement learning method used in this paper—projective simulation (PS) introduced by Briegel and De las Cuevas (2012).

### 5.1. Perceptual States

Let **e** ∈ *A* × *E* be the complete physical state of the environment. In practice, it is impossible to estimate **e**. However, only a facet (e.g., a books orientation) of **e** is required to successfully perform a task. We use haptic exploration (e.g., sliding) in order to estimate the relevant fraction of **e**. A predefined set of sensing behaviors *S* is used to gather information. For many tasks only one sensing behavior *s* ∈ *S* is required to estimate the relevant information, e.g., the book's orientation can be determined by sliding along the surface. While the sensing behavior *s* is executed, a multi-dimensional sensor data time series (e.g., fingertip sensor data) *M* = {**t**_τ_} of duration *T* with τ ∈ [1, …, *T*] is measured. This time series is not the result of a deterministic process but follows an unknown probability distribution *p*(*M* | **e**, *s*).

In general, in every state **e** ∈ *A* × *E* potentially a different behavior has to be executed in order to fulfill a task successfully, e.g., how to grasp an object depends on the object pose. However, in many manipulation problems, similar states require a similar or even the same action. In these cases the state space can be divided into discrete classes *e*, e.g., the four orientations of a book in the book grasping task. We call such a class a *perceptual state*, denoted $e\in E_{\sigma }^{s}$. Note that the perceptual state space $E_{\sigma }^{s}$ is not to be confused with the state space of environment *E*. The probability *p*(*e* | *M, s*, σ) of a perceptual state *e* to be present depends on the measured sensor data *M*, the sensing behavior *s* and the skill σ for which the sensing action *s* is used, e.g., poking in book grasping means something different than in box opening. The perceptual state spaces of two sensing behaviors *s, s*′ ∈ *S* can coincide, partly overlap or be distinct, e.g., sliding along the surface allows the robot to estimate the orientation of a book, whereas poking does not.

### 5.2. Projective Simulation

Projective simulation (PS) introduced by Briegel and De las Cuevas (2012) is a framework for the design of intelligent agents and can be used, amongst other applications, for reinforcement learning (RL). PS was shown to exhibit competitive performance in several reinforcement learning scenarios ranging from classical RL problems to adaptive quantum computation (Melnikov et al., 2014, 2015; Mautner et al., 2015; Tiersch et al., 2015). It is a core component of our method and was chosen due to structural advantages, conceptual simplicity and good extensibility. We briefly describe the basic concepts and the modifications applied in this paper. A detailed investigation of its properties can be found in work by Mautner et al. (2015).

Roughly speaking, the PS agent learns the probability distribution *p*(*b* | **λ**, **e**) of executing a behavior *b* (e.g., a preparatory behavior) given the observed sensor data **λ** (e.g., a verbal command regarding which skill to execute) in order to maximize a given reward function *r*(*b*, **λ**, **e**). In this paper, reward is given if Success^σ^(*b* ∘ *b*^σ^(**e**)) = true, given a command λ to execute skill σ in the present environment state **e**. Note that the state **e** is never observed directly. Instead, perceptual states are estimated throughout the skill execution.

**Figure 1:**
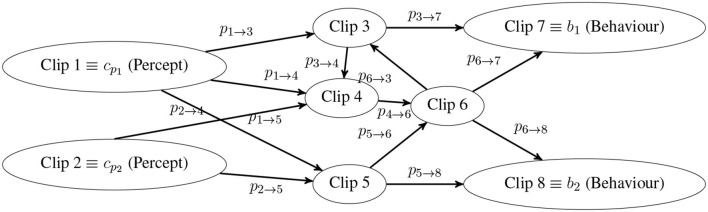
Exemplary sketch of an episodic and compositional memory (ECM). A random walk always starts at a percept clip (e.g., clip 1, clip 2) and ends with a behavior clip (e.g., clip 7, clip 8). A transition *c* → *c*′ from clip *c* to clip *c*′ is done with the probability $p_{c\to c^{\prime }}$. Intermediate clips (e.g., clip 3, clip 6) denote simulation of previous experiences, i.e., behaviors and percepts. The extensions made in this work enable the robot to create transitions between perceptual states on Layer D (i.e., active learning) and to add create new preparatory behaviors on Layer E.

In general, the core of the PS agent is the so-called *episodic and compositional memory* (ECM; first coined in biology by Tulving, 1972). An exemplary sketch of an ECM is shown in Figure 1. It stores fragments of experience, so-called *clips*, and connections between them. Each clip represents a previous experience, i.e., percepts and actions.

The distribution *p*(*b* | **λ**, **e**) is updated after a rollout, i.e., observing a percept, choosing and executing a behavior according to *p*(*b* | **λ**, **e**), and receiving reward from the environment. The distribution *p*(*b* | **λ**, **e**) is implicitly specified by assigning transition probabilities $p_{c\to c^{\prime }}=p\left(c^{\prime }|c\right)$ to all pairs of clips (*c, c*′) (in Figure 1 only transitions with probability $p_{c\to c^{\prime }}\neq 0$ are visualized). Given a certain *percept clip*, i.e., a clip without inbound transitions like clips 1 and 2, the executed *behavior clip*, i.e., a clip without outbound transitions like clips 7 and 8, is selected by a *random walk* through the ECM. A random walk is done by hopping from clip to clip according to the respective transition probabilities until a behavior is reached. Clips are discrete whereas sensor data is typically continuous, e.g., voice commands. A domain-specific input coupler distribution *I*(*c*_*p*_|**λ**, **e**) modeling the probability of observing a discrete percept clip *c*_*p*_ given an observed signal **λ** is required. The distribution *p*(*b* | **λ**, **e**) is given by a random walk through the ECM with

(4) $$\begin{array}{l} p\left(b|\lambda ,\text{e}\right)=\underset{c_{p}}{\sum }\left(I\left(c_{p}|\lambda ,\text{e}\right)\underset{w\in \Lambda \left(b,c_{p}\right)}{\sum }p\left(b|c_{p},w\right)\right) \end{array}$$

where *p*(*b* | *c*_*p*_, *w*) is the probability of reaching behavior *b* from percept *c*_*p*_ via the path *w* = (*c*_*p*_ = *c*_1_, *c*_2_, …, *c*_*K*_ = *b*). The set Λ(*b, c*_*p*_) is the set of all paths from the percept clip *c*_*p*_ to the behavior clip *b*. The path probability is given by

(5) $$\begin{array}{l} p\left(b|c_{p},w\right)=\overset{K-1}{\underset{j=1}{\prod }}p\left(c_{j+1}\;|\;c_{j}\right) \end{array}$$

The agent learns by adapting the probabilities $p_{c\to c^{\prime }}$ according to the received reward (or punishment) *r* ∈ ℝ. The transition probability $p_{c\to c^{\prime }}$ from a clip *c* to another clip *c*′ is specified by the abstract transition weights *h* ∈ ℝ^+^ with

(6) $$\begin{array}{l} p_{c\to c^{\prime }}=p\left(c\;|\;c^{\prime }\right)=\frac{h_{c\to c^{\prime }}}{\sum_{\hat{c}}h_{c\to \hat{c}}} \end{array}$$

After each rollout, all weights $h_{c\to c^{\prime }}$ are updated. Let *w* be a random walk path with reward *r*^(*t*)^ ∈ ℝ at time *t*. The transition weights are updated according to

(7) $$\begin{array}{l} h_{c\to c^{\prime }}^{t+1}=\text{max}\left(1,h_{c\to c^{\prime }}^{t}+\rho \left(c,c^{\prime },w\right)r^{t}\right) \end{array}$$

where ρ(*c, c*′, *w*) is 1 if the path *w* contains the transition *c* → *c*′ and 0 otherwise. Note that in the case of *r* ∈ ℝ^−^, the reward can be considered as punishment. A punishment only has effect in case the initial weight *h*_init_ is >1 (c.f. Table 1) or if an action was rewarded previously, but does not yield a successful skill execution anymore.

**Table 1:** List of free parameters and values used.

**Parameter**	**Name**	**Values**
Skill success reward	*r*(success)	1,000
Skill failure punishment	*r*(failure)	−50
Environment model reward	*r*^env^	10
Skill ECM initial weight	*h*_init_	200
Environment model ECM initial weight	$h_{\text{init}}^{\text{env}}$	1
Stretching factor	α	25
Boredom affinity	β	{0.1, 0.3, 0.5, 0.8, 1.0}
Boredom base	β_base_	0.5
Squashing scale	γ	0.1
Squashing shift	δ	0.95
Balancing factor	ϵ	0.1
Maximum composition path length	*L*_max_	4

In prior work by Melnikov et al. (2018) and Clausen et al. (2019), PS was compared to RL methods like Q-Learning or SARSA. The convergence behavior was found to be similar to these methods. In addition, as PS exhibits parallels to biological agents by incorporating the idea of an episodic and compositional memory (ECM), it was demonstrated to be able to naturally mimic behaviors of real biological agents (c.f. work by Ried et al., 2019). As in this work we aim for drawing parallels to biological agents (c.f. Piaget, 1952), we chose PS over RL approaches like Q-Learning. In particular, Mautner et al. (2015) describe mechanisms for clip composition/merging and clip creation which will be used in this work in creating the environment model and novel behaviors. Additionally, PS allows us to parsimoniously represent the forward (environment) model and the control policy model within the same formalism.

## 6. Skill Learning by Robotic Playing

The following section describes the method for autonomous skill acquisition by autonomous play introduced by Hangl et al. (2016) on which this work is based. The sections 8–10 present extensions that run in parallel and augment autonomous play. In addition, the contribution of this section is to provide a more solid theoretical formulation of the previous approach described by Hangl et al. (2016) such that the connection to the method proposed in this work can be seen.

A new skill is taught to the robot by human-robot interaction. In the book grasping task (section 1), the human teacher shows the robot to grasp the book in a situation in which the book has an optimal orientation, i.e., the spine shows away from the robot such that the robot can lift the book at the spine in order to get its fingers under the book. The teaching can be done by kinesthetic teaching, visual programming or any other robot programming technique. This behavior is the *basic behavior* of the skill and does not work if the book is oriented differently. The robot would just try to lift the book and would open it instead of lifting it. Next, the robot uses its sensing behaviors to explore the book in different states, i.e., the four different orientations. If the human prepares these different situations, she provides prior knowledge to the robot about the semantics of the system without having to know about internals of the robot. This draws a parallel to infant-adult play in which the adult prepares different interesting states of the toy for the child. Alternatively, the robot can prepare different states using its preparatory behaviors, assuming that the execution of a behavior results in a new state. The robot gathers information about the object with its sensing behaviors, e.g., *poking, sliding*, or *pressing*. At this point the robot has no understanding of which sensing behaviors provide the best data and what the semantics of the states actually is; it has no concept of the orientation of the book. By running complete loops of *sensing* → *preparation* → *basic behavior execution* → *reward*, the robot learns which sensing behavior and which preparatory behaviors are most useful. In this example the robot needs to rotate the book before it applies the basic behavior. This way it learns how to grasp the book in any arbitrary orientation. After completion of the learning, the new skill is added the repertoire of preparatory behaviors. For example, the book grasping skill might be useful if the robot is asked to place the book on a shelf.

This example showcases an important difference of our notion of *play* to traditional reinforcement learning methods. The human is involved in the learning loop, but only insofar as it is semantically meaningful. Knowledge about robot internals, such as controllers or sensing capabilities is not required. Human domain knowledge is incorporated into the learning stage in a way that resembles infant play.

### 6.1. ECM for Robotic Playing

**Figure 2:**
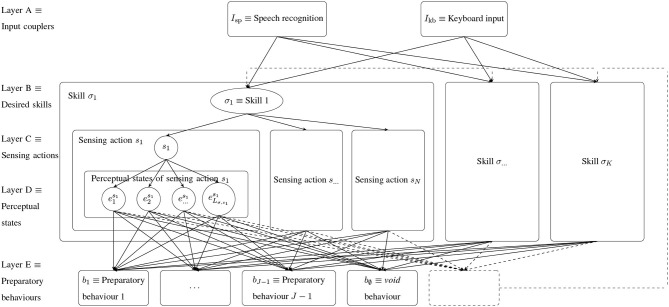
ECM for autonomous robotic playing. For execution a random walk is performed from layer A to layer E. The transition from layer C to layer D is performed by executing the corresponding sensing behavior *s*, measuring the haptic data and using a time series classifier. All other transitions follow Equation (6). The preparatory behavior *b*_∅_ ≡ (*void* behavior) is always in the set of preparatory behaviors. The dashed box and lines refer to skills used as preparatory behaviors in order to build skill hierarchies. After preparation, the *basic* behavior *b*^σ^ corresponding to the desired skill σ is executed. The active learning approach presented in this paper enables the robot to perform transitions between clips on layer D. The behavior composition extension helps the robot to add new preparatory behavior compositions in layer E. The ECM shown in this figure is independent of the environment model and will only be influenced (e.g., by adding new clips) in case the environment model becomes more mature.

A skill σ is executed by a random walk through the layered ECM shown in Figure 2, where each clip corresponds to executing the corresponding behavior. It consists of the following layers:

1. The input coupler maps user commands stating which skill to execute to the corresponding skill clip.
2. Skill clips σ represent skills the robot is able to perform. If the user commands the robot to execute a certain skill (e.g., grasping), the corresponding skill clip will be excited. From there, the skill execution starts by performing a random walk (c.f. section 5.2).
3. Sensing behavior clips *s* ∈ *S* correspond to the execution of sensing behaviors. All skills share the same sensing behaviors.
4. Perceptual state clips $e\in E_{\sigma }^{s}$ correspond to perceptual states under the sensing behavior *s* for the skill σ. Note that the perceptual states are different for each skill/sensing behavior pair (σ, *s*) and typically do not have the same semantics, e.g., the states under sensing behavior *s* ∈ *S* might identify the object pose, whereas the states under *s*′ ∈ *S* might denote the object's concavity. The perceptual states (and the matching sensor data) are created during the creation of the haptic database (c.f. section 6.2.1). During database creation, similar to human play, different situations are presented and the robot actively explores the object with its sensing behaviors.
5. Preparatory behavior clips *b* correspond to behaviors which can be atomic (solid transitions) or other trained skills (dashed transitions). Since the basic behavior *b*^σ^ of a skill was shown to the robot in one perceptual state, there is at least one state that does not require preparation. Therefore, the *void* behavior *b*_∅_, in which no preparation is done, is in the set of behaviors.

The robot holds the sets of skills {σ = (*b*^σ^, Success^σ^)}, sensing behaviors *S* (e.g., sliding, poking, pressing) and preparatory behaviors *B* (e.g., pushing). A skill is executed by performing a random walk through the ECM and by performing the actions along the path. The idle robot waits for a skill execution command λ which is mapped to skill clips in Layer B by coupler functions, e.g., *I*_kb_ and *I*_sp_ mapping a keyboard input/voice commands to the desired skill clip σ. A sensing behavior *s* ∈ *S* is chosen and executed according to the transition probabilities and a sensor data time series *M* is measured. The perceptual state $e\in E_{\sigma }^{s}$ is estimated from *M*. This transition is done deterministically by a classifier and not randomly as in the steps before. Given the perceptual state *e*, the environment is prepared by executing a behavior *b* ∈ *B*. Finally, the basic behavior *b*^σ^ is executed. If a basic behavior of a skill requires an object to be grasped, only the sensing behavior *weighing* is available in order to estimate whether an object is grasped. We stress that this is only a restriction enforced due to practical considerations and is not required in principle.

### 6.2. Skill Training

A novel skill σ = (*b*^σ^, Success^σ^) is trained by providing the basic behavior *b*^σ^ for a narrow range of situations, e.g., by hard coding or learning from demonstration (Atkeson and Schaal, 1997; Lopes et al., 2007; Asfour et al., 2008; Argall et al., 2009; Konidaris et al., 2011; Kormushev et al., 2011; Hangl et al., 2015). The domain of applicability is extended by learning:

Problem A: which sensing behavior should be used to estimate the relevant perceptual state;
Problem B: how to estimate the perceptual state from haptic data;
Problem C: which preparatory behavior helps to achieve the task in a given perceptual state.

The skill ECM (Figure 2) is initialized in a meaningful way (sections 6.2.1, 6.2.2) and afterwards refined by executing the skills and collecting rewards, i.e., *autonomous play*.

#### 6.2.1. Haptic Database Creation

In a first step, the robot creates a haptic database by exploring how different perceptual states “feel,” (c.f. problem 2). It performs all sensing behaviors *s* ∈ *S* several times in all perceptual states *e*^*s*^, acquires the sensor data *M* and stores the sets {(*e*^*s*^, *s*, {*M*})}. With this database the distribution *p*(*e* | *M, s*, σ) (section 5.1) can be approximated and a perceptual state classifier is trained. The haptic database size scales with the number of sensing behaviors and perceptual states. However, experience from Hangl et al. (2016) has shown that a small number of sensing behaviors is sufficient to measure the relevant states of a wide range of tasks. Moreover, many tasks can be solved with a small number of perceptual states.

There are two ways of preparing different perceptual states. Either the supervisor prepares the different states (e.g., all four book poses) or the robot is provided with information on how to prepare them autonomously (e.g., *rotate by 90*° produces all poses). In the latter case the robot assumes that after execution of the behavior a new perceptual state *e*′ is present and adds it to the haptic database. This illustrates three important assumptions: The state $e^{s}\in E_{\sigma }^{s}$ is invariant under the sensing behavior *s* ∈ *S* (e.g., the book's orientation remains the same irrespective of how often *sliding* is executed) but not under preparatory behaviors *b* ∈ *B* (e.g., the book's orientation changes by using the *rotate 90*° behavior), which yields

(8) $$\begin{array}{l} e^{s}\overset{s}{\to }e^{s} \end{array}$$

(9) $$\begin{array}{l} e^{s}\overset{b}{\to }e^{\prime s} \end{array}$$

Further *e*^*s*^ is only invariant to the sensing behavior *s* but not necessarily to any other sensing behavior (e.g., sliding softly along a tower made of cups does not change the position of the cups whereas poking from the side may cause the tower to collapse):

(10) $$\begin{array}{l} e^{s}\overset{s}{\to }e^{s}\overset{s}{\to }\ldots \overset{s}{\to }e^{s}\overset{s^{\prime }}{\to }e^{s^{\prime }}\overset{s}{\to }e^{\prime s} \end{array}$$

#### 6.2.2. ECM Initialization

The ECM in Figure 2 is initialized with the uniform transition weights *h*_init_ except for the weights between layers B and C. These weights are initialized such that the agent prefers sensing behaviors *s* ∈ *S* that can discriminate well-between their environment states $e^{s}\in E_{\sigma }^{s}$. After the generation of the haptic database the robot performs cross-validation for the perceptual state classifier of each sensing action *s* ∈ *S* and computes the average success rate *r*_*s*_. A *discrimination score* *D*_*s*_ is computed by

(11) $$\begin{array}{l} D_{s}=\exp\left(\alpha r_{s}\right) \end{array}$$

with the free parameter α called *stretching factor*. The higher the discrimination score, the better the sensing action can classify the corresponding perceptual states. Therefore, sensing behaviors with a high discrimination score should be preferred over sensing behaviors with a lower score. The transition weights between all pairs of the skill clip σ and the sensing behavior clips *s* ∈ *S*, i.e., the connections between layers B and C, are initialized with *h*_σ → *s*_ = *D*_*s*_. This initialization biases the ECM to prefer sensing behaviors which can differentiate well-between perceptual states. In case uniform weights are used learning will converge slower. Due to the semantically meaningful perceptual states, weights between other layers (e.g., between the perceptual state layer and the preparatory behavior layer) could be initialized if a teacher provides initial guesses for useful preparatory behaviors or by observing actions of other agents. However, in this work we focus on learning these weights by autonomous play. We use a C-SVM classifier implemented in LibSVM (Chang and Lin, 2011) for state estimation.

#### 6.2.3. Extending the Domain of Applicability

**Figure 3:**
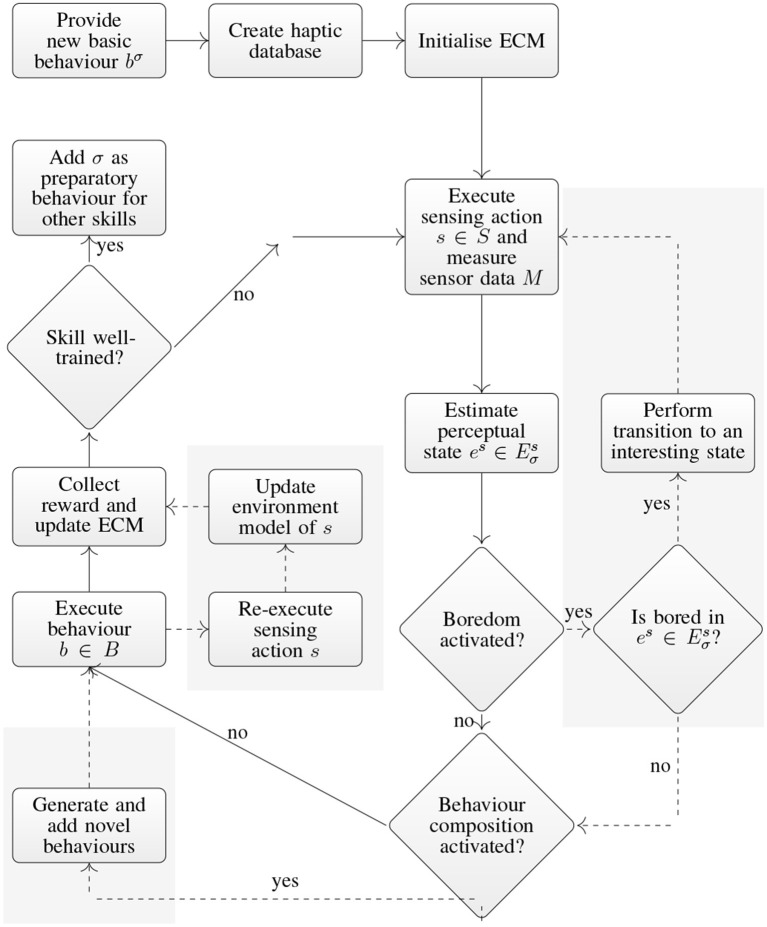
Flow chart of the skill training procedure. A novel skill is trained by showing a new basic behavior. The robot extends the domain of applicability by playing the object, i.e., by performing a random walk through the network shown in Figure 2. The solid lines indicate the behavior of the basic approach (Hangl et al., 2016). The shaded areas and dashed lines show the proposed extensions, i.e., *training of an environment model, boredom*, and *composite behavior generation*.

The domain of applicability of a skill σ is extended by running the PS as described in section 5.2 on the ECM in Figure 2. The robot collects reward after each rollout and updates the transition probabilities accordingly. Skills are added as preparatory behaviors of other skills as soon as they are well-trained, i.e., the average reward $\bar{r}$ over the last *t*_thresh_ rollouts reaches a threshold $\bar{r}\geq r_{\text{thresh}}$. This enables the robot to create increasingly complex skill hierarchies. The complete training procedure of a skill σ is shown in Figure 3. Only the non-shaded parts and solid transitions are available in this basic version.

A key aspect of our method is the analogy to human play. The main idea is that a human teacher plays with the robot in a sense that she is interacting with it while the robot explores the object and the given task. The following stages in Figure 3 require interaction with the human teacher:

- *Provide new basic behavior* *b*^σ^: The human teacher provides a simple skeleton of how to solve a skill in a specific situation. This can be done by simple kinesthetic teaching (by non-experts), by providing sophisticated state-of-the-art learning methods or by simple hard-coding [e.g., with the simple visual programming language by Hangl et al. (2017)].
- *Create haptic database*: The human teacher selects the objects used by the robot to learn the skill. The teacher also provides the robot with different, semantically-meaningful perceptual states, allowing the robot to explore the task in diverse situations. Moreover, certain situations may have to be provided several times if the robot destroys the perceptual states or is not able to reproduce them by itself. We stress the parallel to early-stage human play where a teacher selects objects and guides the infant by preparing situations that may be helpful in learning.
- *Collect reward and update ECM*: The robot requires rewards in order to update the ECM probabilities after it has executed a certain sequence of behaviors. The reward is provided by the teacher.

In this way, teaching can be done by non-experts without particular robotics knowledge by a simple way of interacting with the robot. The teacher does not have access to internals of the robot (sensors, actuators) but provides input to the robot in a simple manner while the robot will use its own repertoire of (sensing and preparatory) behaviors and previously-trained skills, including the internal sensing used by these behaviors.

## 7. Properties and Extensions

An important property of our methods is that state-of-the-art research on object manipulation can be embedded by adding the controllers to the set of behaviors. Algorithms for specific problems (e.g., grasping, pushing by Whitney, 1987; Li, 1997; Omrcen et al., 2009; Mülling et al., 2013; Krivic et al., 2016) can be re-used in a bigger framework that orchestrates their interaction.

Hangl et al. (2016) have shown that typical scenarios can be done by using a small number of sensing behaviors/perceptual states/preparatory behaviors (e.g., 4/4–10/5–10). This enables the robot to learn skills without an environment model. Further, the robot still learns fast while preserving the ability to learn quite complex skills autonomously. However, the lack of an environment model can be both an advantage and a disadvantage. Testing a hypothesis directly on the environment enables the robot to apply behaviors outside of the intended context [e.g., a book flipping behavior might be used to open a box (Hangl et al., 2016)]. This is hard to achieve with model-based approaches if the modeled domain of a behavior cannot properly represent the relevant information. On the other hand, the lack of reasoning abilities limits the learning speed and the complexity of solvable problems. We overcome this problem by additionally learning an environment model from information acquired during playing. The robot learns a distribution of the effects of behaviors on given perceptual states by re-estimating the state after execution. We use the environment model for two purposes: *active learning* and *compositional generation of novel preparatory behaviors*.

The basic version intrinsically assumes that all required preparatory behaviors are available. This constitutes a strong prior and limits the degree of autonomy. We weaken this requirement by allowing the robot to generate potentially useful combinations of behaviors. These are made available for the playing system which tries them out. Further, experiments will show that the overall learning speed is decreased by performing rollouts in situations that were already solved before. This is because the rollout could be spent on situations where there is still something to learn. We use the environment model to implement *active learning*. Instead of asking a supervisor to prepare interesting situations, the robot prepares them by itself.

## 8. Learning an Environment Model

The *environment model* predicts the effect, i.e., the resulting perceptual state, of a behavior on a given perceptual state. An environment model is the probability distribution *p*(*e*^′*s*^ | *e*^*s*^, *b*, σ) where $e^{s},e^{\prime s}\in E_{\sigma }^{s}$ are perceptual states of the sensing behavior *s* ∈ *S* for a skill σ, and *b* ∈ *B* is a behavior. It denotes the probability of the transition $e^{s}\overset{b}{\to }e^{\prime s}$. The required training data is acquired by re-executing the sensing behavior *s* after applying the behavior *b*, c.f. shaded center part in Figure 3. Given a playing sequence $\sigma \overset{s}{\to }e^{s}\overset{b}{\to }e^{\prime s}$ (c.f. Figure 3) the effect can be observed by re-executing *s* with

(12) $$\begin{array}{l} \sigma \overset{s}{\to }e^{s}\overset{b}{\to }e^{\prime s}\overset{s}{\to }e^{\prime s} \end{array}$$

The assumptions in Equations (8)–(9) forbid to additionally execute other sensing behaviors *s*′ ∈ *S* without influencing the playing based method. This limitation prevents the robot from learning more complex environment models as done in related work by Barto et al. (2004), Stoytchev and Arkin (2004), Oudeyer et al. (2007), Schembri et al. (2007), Baranes and Oudeyer (2009), and Ugur and Piater (2016), e.g., capturing transitions between perceptual states of different sensing behaviors. However, the purpose of the environment model is not to perform precise plans but to feed the core model-free playing component with suggestions for new behaviors to try out.

We represent the distribution *p*(*e*^′*s*^ | *e*^*s*^, *b*, σ) by another ECM for each skill—sensing behavior pair (σ, *s*). The percept clips consist of pairs (*e*^*s*^, *b*) of perceptual states $e^{s}\in E_{\sigma }^{s}$ and preparatory behaviors *b* ∈ *B*. The target clips are the possible resulting states $e^{\prime s}\in E_{\sigma }^{s}$. The environment model is initialized with uniform weights $h_{\left(e^{s},b\right)\to e^{\prime s}}^{\text{env}}=1$. When a skill σ is executed using the path in Equation (12), a reward of *r*^env^ ∈ ℝ^+^ is given for the transition

(13) $$\begin{array}{l} \left(e^{s},b\right)\to e^{\prime s} \end{array}$$

and the weights are updated accordingly (c.f. Equation 7). When a novel preparatory behavior *b*_*K*+1_ is available for playing, e.g., a skill is well-trained and is added as a preparatory behavior, it is included into the environment models for each skill-sensing behavior pair (σ, *s*) by adding clips (*e*^*s*^, *b*_*K*+1_) for all states $e^{s}\in E_{\sigma }^{s}$ and by connecting them to all $e^{\prime s}\in E_{\sigma }^{s}$ with the uniform initial weight $h_{\text{init}}^{\text{env}}=1$.

## 9. Autonomous Active Learning

In the basic version an optimal selection of observed perceptual states is required in order to learn the correct behavior in all possible states, i.e., in a semi-supervised setting a human supervisor should mainly prepare unsolved perceptual states. This would require the supervisor to have knowledge about the method itself and about the semantics of perceptual states, which is an undesirable property. Instead, we propose to equip the robot with the ability to reject perceptual states in which the skill is well-trained already. In an autonomous setting, this is not sufficient as it would just stall the playing. The robot has to prepare a more interesting state autonomously. We propose to plan state transitions by using the environment model in order to reach states which (i) are *interesting* and (ii) can be prepared with high confidence. We can draw a loose connection to human behavior. In that spirit, we call the rejection of well-known states *boredom*.

### 9.1. Boredom

The robot may be *bored* in a given perceptual state, if it is *confident* about the task solution, i.e., if the distribution of which preparatory behavior to select is highly concentrated. In general, every function reflecting uncertainty can be used. We use the normalized Shannon entropy to measure the confidence in a perceptual state $e\in E_{\sigma }^{s}$, given by

(14) $$\begin{array}{l} \begin{array}{c} {\hat{H}}_{e}=\frac{H\left(b|e\right)}{H_{\text{max}}}=-\frac{\sum_{b^{\prime }\in B}p\left(b=b^{\prime }|e\right)\log_{2}p\left(b=b^{\prime }|e\right)}{\log_{2}J} \end{array} \end{array}$$

where *J* is the number of preparatory behaviors. If the entropy is high, the robot either has not learned anything yet (and therefore all the transition weights are close to uniform) or it observes the degenerate case that all preparatory behaviors deliver (un)successful execution (in which case there is nothing to learn at all). If the entropy is low, few transitions are strong, i.e., the robot knows well how to handle this situation. We use the normalized entropy to define the probability of being bored in a state $e\in E_{\sigma }^{s}$ with

(15) $$\begin{array}{l} p\left(\text{bored}=true|e\right)=\min\left(\beta \left(1-{\hat{H}}_{e}\right)+\beta_{\text{base}},1.0\right) \end{array}$$

The constant β ∈ [0, 1] defines how *affine* the agent is to boredom and β_base_ provides a *boredom base*. The robot samples according to *p*(bored | *e*) and decides on whether to refuse the execution.

### 9.2. Transition Confidence

If the robot is bored in a perceptual state $e^{\prime }\in E_{\sigma }^{s}$, it autonomously tries to prepare a more interesting state $ê\in E_{\sigma }^{s}$. This requires the notion of a *transition confidence* for which the environment model can be used. We aim to select behaviors conservatively which allows the robot to be certain about the effect of the transition. We do not use the probability of reaching one state from another directly, but use a measure considering the complete distribution *p*(*e* | *e*′, *b*). By minimizing the normalized Shannon entropy, we favor deterministic transitions. For each state-action pair (*e*′, *b*) we define the transition confidence $\nu_{e^{\prime }b}^{s}$ by

(16) $$\begin{array}{l} \nu_{e^{\prime }b}^{s}=1-\frac{H\left(e|\left(e^{\prime },b\right)\right)}{H_{\text{max}}}=1-\frac{H\left(e|\left(e^{\prime },b\right)\right)}{\log_{2}L_{\sigma ,s}} \end{array}$$

where $e^{\prime }\in E_{\sigma }^{s}$, *b* ∈ *B*, and *L*_σ,*s*_ is the number of perceptual states under the sensing behavior *s* ∈ *S*, i. e. the number of children of the clip (*e*′, *b*). In contrast to the entropy computed in section 9.1, the transition confidence is computed on the environment model. The *successor function* su(*e, b*) returns the most likely resulting outcome of executing behavior *b* in a perceptual state $e\in E_{\sigma }^{s}$ and is defined by

(17) $$\begin{array}{l} \text{su}\left(e,b\right)=\text{arg }\underset{e^{\prime }}{\max}p_{\left(e,b\right)\to e^{\prime }} \end{array}$$

In practice, single state transitions are not sufficient. For paths $e=e_{1}^{s}\overset{b_{1}}{\to }e_{2}^{s}=\text{su}\left(e_{1}^{s},b_{1}\right)\overset{b_{2}}{\to }\ldots \overset{b_{L-1}}{\to }\text{su}\left(e_{n_{L-1}}^{s},b_{L-1}\right)=e_{L}^{s}=e^{\prime }$ of length *L* we define the transition confidence with

(18) $$\begin{array}{l} \nu_{e\text{b}}^{s}=\overset{L-1}{\underset{l=1}{\prod }}\nu_{e_{l}b_{l}}^{s} \end{array}$$

where the vector **b** = (*b*_1_, *b*_2_, …, *b*_*L*−1_) denotes the sequence of behaviors. This is equivalent to a greedy policy, which provides a more conservative estimate of the transition confidence and eliminates consideration of transitions that could occur by pure chance. A positive side effect is the efficient computation of Equation (18). Only the confidence of the most likely path is computed instead of iterating over all possible paths. The path **b** is a behavior itself and the successor is given by $\text{su}\left(e,\text{b}\right)\;=\;\text{su}\left(e_{n_{L-1}}^{s},b_{L-1}\right)$.

### 9.3. Active Learning

If the robot encounters a boring state $e\in E_{\sigma }^{s}$, the goal is to prepare the most interesting state that can actually be produced. We maximize the *desirability function* given by

(19) $$\begin{array}{l} \left(\text{b},L\right)=\text{arg }\underset{\text{b},L}{\max}\left[{\hat{H}}_{\text{su}\left(e,\text{b}\right)}\nu_{e\text{b}}+\frac{\epsilon }{\text{cost}\left(\text{b}\right)}\right] \end{array}$$

where Ĥ_su(*e*,**b**)_ is the entropy of the expected final state and ν_*e***b**_ is the confidence of reaching the final state by the path **b**. The *balancing factor* ϵ defines the relative importance of the desirability and the path cost. The path cost cost(**b**) can be defined by the length of the path *L*, i.e., penalizing long paths, or, for instance, by the average execution time of **b**. Equation (19) balances between searching for an interesting state while making sure that it is reachable. In practice it can be optimized by enumerating all paths of reasonable length, e.g., *L* < *L*_max_, with typical values of *L*_max_ ≤ 4.

The basic method is extended by sampling from the boredom distribution after the state estimation. If the robot is bored, it optimizes the desirability function and executes the transition to a more interesting state if it is more interesting than the current one. This is followed by restarting the skill execution with boredom turned off in order to avoid boredom loops (c.f. right shaded box in Figure 3).

## 10. Composite Behavior Generation

In many cases, the required preparatory behavior is a combination of other available behaviors, e.g., *rotate 180*° ≡ *rotate 90*° + *rotate 90*°. Without using some sort of intelligent reasoning, the space of concatenated behaviors explodes and becomes intractable. However, any sequence of behaviors that transfers the current unsolved state to a target state, i.e., a state in which the solution is already known, is potentially useful as a compound behavior itself. Sequences can be generated by planning transitions to target states. If the robot is bored, it uses active learning, if not, the situation is not solved yet and novel compound behaviors might be useful. A perceptual state $e_{\text{target}}\in E_{\sigma }^{s}$ is a target state if the strongest connection to a child clip *b*_target_ in the playing ECM (Figure 2) exceeds a certain threshold. If there exists a path $e^{s}\overset{\bar{\text{b}}}{\to }e_{\text{target}}$ from the current perceptual state $e^{s}\in E_{\sigma }^{s}$ to a target state *e*_target_, the sequence $\text{b}=\left(\bar{\text{b}},su\left(e_{\text{target}}\right)\right)=\left(b_{1},\ldots ,b_{L},su\left(e_{\text{target}}\right)\right)$ is a candidate for a novel behavior. The robot is “*curious*” about trying out the novel compound behavior **b**, if the transition confidence $\nu_{e^{s}\text{b}}$ and the probability *p*_*e*_target_→*b*_target__ of the state actually being a real target state are both high. This is measured by the *curiosity score* of the compound behavior given by

(20) $$\begin{array}{l} \text{cu}\left(e^{s},\text{b}\right)=\nu_{e^{s}\bar{\text{b}}}p_{e_{\text{target}}\to b_{\text{target}}} \end{array}$$

The factor *p*_*e*_target_→*b*_target__ reduces the score in case *e*_target_ is a target state with low probability. This can happen if in previous rollouts all other behaviors were executed and were punished. We use a probability instead of a confidence value to allow behavior composition even in early stages where a target state was not identified with a high probability.

The compound behavior **b** with the highest score is added as novel behavior *b*_*J*+1_ with the probability given by squashing the curiosity score into the interval [0, 1] with

(21) $$\begin{array}{l} p_{\text{add}}\left(b_{J+1},e^{s}\right)=\text{sig}\left[\gamma \text{ cu}\left(e^{s},\text{b}\right)+\delta \right] \end{array}$$

where sig is the logistic sigmoid. The parameters γ, δ define how conservatively novel behavior proposals are created. The novel behavior *b*_*J*+1_ is added as preparatory behavior for all perceptual states under the current skill σ with the weights

(22) $$\begin{array}{l} \begin{array}{l} h_{e\to b_{J+1}}=\{\begin{array}{ll} h_{\text{init}}\left[1+\text{cu}\left(e^{s},\text{b}\right)\right] & \text{if}e=e^{s}\\ h_{\text{init}} & \text{otherwise} \end{array} \end{array} \end{array}$$

**Figure 4:**
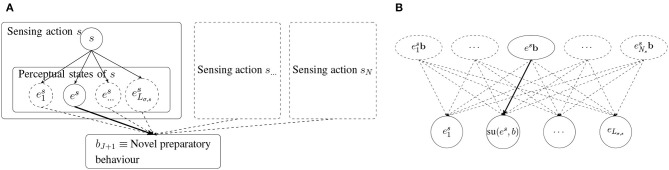
Insertion of the novel compound behavior *b*_*J*+1_ = **b**, generated in the current perceptual state $e^{s}\in E_{\sigma }^{s}$, to the playing ECM of skill σ (left) (c.f. Figure 2), and the environment models of the used sensing behavior *s* ∈ *S* (right), respectively. **(A)** The behavior *b*_*J*+1_ is added as child clip to all perceptual states except for the current state *e*^*s*^ with the initial weight *h*_init_ (dashed lines). In this case it is added with a higher weight proportional to the curiosity score (solid line) (c.f. Equation 22). The connections to all other preparatory behaviors are omitted in this figure. **(B)** All pairs (*e, b*_*J*+1_) of perceptual states $e\in E_{\sigma }^{s}$ and the behavior *b*_*J*+1_ = **b** are added. The weights are chosen according to Equation (23) (case 1: solid line, case 2: dashed lines). Note that case 1 only applies for the currently used sending action *s* ∈ *S*.

It is added with at least the initial weight *h*_init_, but increased proportional to the curiosity score for the current perceptual state $e^{s}\in E_{\sigma }^{s}$ (c.f. Figure 4A). The novel behavior is also inserted to the environment model of all sensing behaviors *s* ∈ *S*. For each perceptual state $e\in E_{\sigma }^{s}$, a clip (*e, b*) is added and connected to the clips $e^{\prime }\in E_{\sigma }^{s}$ in second layer with the weights

(23) $$\begin{array}{l} \begin{array}{l} h_{\left(e,b\right)\to e^{\prime }}=\{\begin{array}{ll} h_{\text{min}}\left(b\right) & \text{if}e=e^{s},e^{\prime }=\text{su}\left(e^{s},b\right),b=b_{J+1}\\ h_{\text{init}}^{\text{env}} & \text{otherwise} \end{array} \end{array} \end{array}$$

where *h*_min_(*b*_*J*+1_) = *h*_min_(**b**) is the minimum transition value on the path **b** through the environment model, following the idea that a chain is only as strong as its weakest link. The weights of all other transitions are set to the initial weight $h_{\text{init}}^{\text{env}}$ (c.f. Figure 4B).

## 11. Experiments

We evaluate our method using a mix of simulated and real-world experiments. Our real-world experiments cover a wide range of skills to show the expressive power. We show how skill hierarchies are created within our framework. Success statistics of the single components (sensing accuracy, success rate of preparatory behaviors, success rate of basic behaviors) were used to assess the convergence behavior by simulation. Table 1 lists the used parameter values. We execute all skills and behaviors in impedance mode in order to prevent damage to the robot. Further, executed behaviors are stopped if a maximum force is exceeded. This is a key aspect for model-free playing, which enables the robot to try out arbitrary behaviors in arbitrary tasks.

### 11.1. Experimental Setup

**Figure 5:**
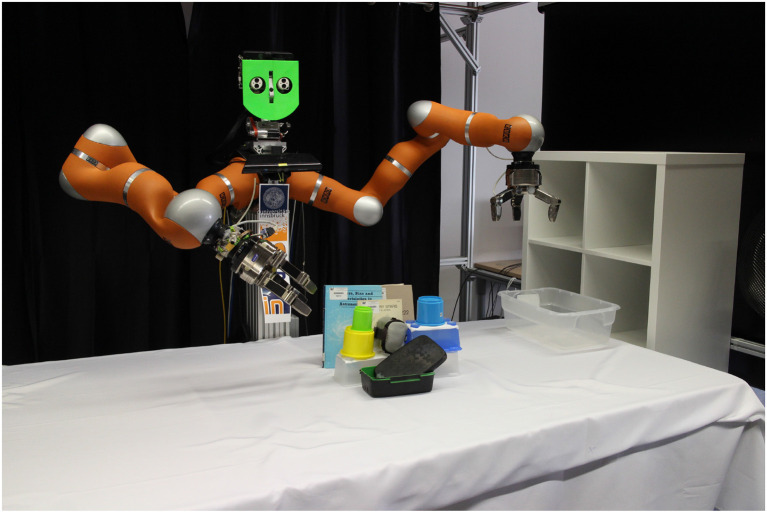
Robot setting and used objects. The hardware included a Kinect, two KUKA LWR 4+ and two Schunk SDH grippers. The objects used for the trained tasks were books of different dimensions and cover types, an IKEA shelf and boxes, and selected objects of the YCB object and model set (Calli et al., 2015).

The robot setting is shown in Figure 5. For object detection a Kinect mounted above the robot is used. All required components and behaviors are implemented with the *kukadu* robotics framework^1^. The perceptual states are estimated from joint positions, Cartesian end-effector positions, joint forces and Cartesian end-effector forces/torques. Objects are localized by removing the table surface from the point cloud and fitting a box by using PCL. Four controllers implement the available preparatory behaviors:

- *Void behavior*: The robot does nothing.
- *Rotation*: The object is rotated by a circular finger movement around the object's center. The controller can be parameterized with the approximate rotation angle. The rotation behaviors incorporate vision feedback loops in order to achieve a success rate close to 100% with the used objects.
- *Flip*: The object is squeezed between the hands and one hand performs a circle with the radius of the object in the XZ-plane which yields a vertical rotation.
- *Simple grasping*: The gripper is positioned on top of the object and the fingers are closed.

The haptic database consists of at least 10 samples per perceptual state. Before sensing, the object is pushed back to a position in front of the robot. We use four sensing behaviors:

- *No Sensing*: Some tasks do not require any prior sensing and have only one state. The discrimination score is computed with a success rate of *r*_*s*_ = 0.5 (c.f. Equation 11).
- *Slide*: A finger is placed in front of the object. The object is pushed toward the finger with the second hand until contact or until the hands get too close to each other (safety reasons). Sensing is done by bending the finger.
- *Press*: The object is pushed with one hand toward the second hand until the force exceeds a certain threshold.
- *Poke*: The object is poked from the top with a finger.
- *Weigh*: Checks a successful grasp by measuring the *z*-component of the Cartesian force. The perceptual states are fixed, i.e., not grasped/grasped.

For all skills described below, there was at least one sensing behavior that yielded an average success rate *r*_*s*_ of at least 95%.

All described behaviors were optimized for maximum success rate instead of high execution speed. This is also possible as a key concept of our method is that sub-behaviors can be arbitrarily complex while hiding all complexity to the outside. In addition, the basic behaviors only have to work on a narrow range of situations, which makes it much easier to implement these with a high success rate. Objects are always pushed back in front of the robot after executing a behavior in order to increase success rate and reduce complexity in the behavior controllers. Sensing behaviors typically required an execution time of about 2 min, the execution of the previously described preparatory behaviors took between 1 and 6 min (c.f. videos^2^^,^^3^). The execution time of basic behaviors ranged from 1 to 3 min. A complete random walk through the skill ECM took between 4 and 11 min. Typical skill training required around 6 h, however, the training time could be significantly improved by optimizing for more execution speed.

### 11.2. Real-World Tasks

We demonstrate the generality of our method in several scenarios. Each skill can use the described preparatory behaviors, and additionally, the skills trained before. A skill is considered to be trained successfully, if the success rate over a sliding window is >0.9. If not stated otherwise, all basic behaviors are dynamic movement primitives (DMPs) (Schaal, 2006) trained by kinesthetic teaching. A video of the trained skills including a visualization of the generated skill hierarchies can be viewed online and is included in the Supplementary Material of this paper^2^. Note that only the skills and behaviors with non-zero probabilities are shown in the hierarchies. The training of skills does not look different to the training in the basic method except for the additional execution sensing behavior after the performed preparation^3^. Some skills were already implemented with the original approach by Hangl et al. (2016), in which a citation is added. These skills were extended, e.g., by relaxing restrictions using the novel approach presented in this work.

#### 11.2.1. Simple Placement

The task is to pick an object and place it in an open box on the table. The basic behavior is a DMP that moves the grasped object to the box, where the hand is opened. In this case, the used sensing behavior is *weigh* (c.f. section 6.1), to determine whether or not an object is in the hand already. After training the *simple grasp*/*nothing* behavior is used if the object is not grasped/grasped, respectively.

#### 11.2.2. Book Grasping

**Figure 6:**
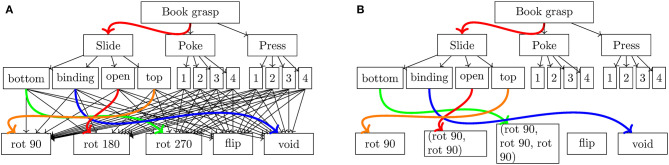
Comparison of the results on a book grasping task between the original approach by Hangl et al. (2016) **(A)** and the novel extension presented in this work **(B)**. **(A)** Resulting ECM after training with the original approach by Hangl et al. (2016) on a book grasping task. All required preparatory behaviors have to be available at the beginning of the training process. As the training is only done model-free, training converges slower (c.f. section 11.3) and no preparatory behaviors can be generated when needed. The colored edges denote strong connections with high transition probabilities. **(B)** Exemplary resulting ECM of our novel approach on a book grasping task. At the beginning of the training process only the behaviors *rotate 90, flip* and *void* were available. The behaviors shown in brackets were created using behavior composition. Note that due to the probabilistic nature of Equation (21) other behvaiors could be created like *rotate 90, void, rotate 90*. For the sake of readability, the weak connections were omitted.

The basic behavior grasps a book as described in section 1. The perceptual states are the four orientations of the book. After training, the robot identified *sliding* as a useful sensing behavior to estimate the book's rotation. The rotation could be determined as the 4 different sides had different haptic properties. The skill is trained with and without using behavior composition. Without behavior composition, the available preparatory behaviors are the *void* behavior, *rotate 90*°, *rotate 180*°, *rotate 270*°, and *flip*. The *rotation* and *void* behaviors are used for different rotations of the book. This task was already solvable with the method by Hangl et al. (2016), but with slower convergence (c.f. section 11.3) and with all preparatory behaviors necessarily being present. When the behaviors *rotate 180*° and *rotate 270*° are removed from the set of preparatory behaviors, the task is *not solvable* with the original method but with the novel extensions presented in this work. The robot creates these behaviors by composing *rotate 90*° two/three times, respectively. A comparison between the original approach and the extension with behavior composition is shown in Figure 6.

#### 11.2.3. Placing Object in a Box

The task is to place an object inside a box that can be closed. The basic behavior is to grasp an object from a fixed position and drop it into an open box (c.f. Hangl et al. 2016). The perceptual states determine whether the box is *open* or *closed*. After training, the robot identifies *poke* as a good sensing behavior. The *flip* behavior is used to open the closed box and the *void* behavior is used if the box is open. This showcases a strong advantage of model-free playing as behaviors can be used beyond their initial purpose. The *flip* behavior is implemented to flip an object but later proves useful to open the box in the *box placement* task. This task is an example where the active learning approach does not increase training speed in all cases, as not all unsolved perceptual states can be produced from solved ones, e.g., if the *closed* state is solved before the *open* state. The robot is only able to prepare the transition *closed* $\overset{flip}{\to }$ *open*. The transition *open* → *closed* requires to close the cover, which is not among the available behaviors. Moreover, behavior composition does not provide any improvement in this scenario as all required basic behaviors are already present.

#### 11.2.4. Complex Grasping

The task is to grasp objects of different sizes. We use the *void* behavior as the skill's basic behavior. This causes the robot to combine behaviors without additional input from the outside. The perceptual states correspond to small and big objects. As sensing behavior, *sliding* ($r_{s}^{\text{sliding}}\approx 1.0$) is used for estimating the object size for *complex grasping* instead of the expected *pressing* ($r_{s}^{\text{pressing}}\approx 0.9$) from which the object size could be derived from the distance between the hands. The high success rate of *sliding* is unexpected and is an artifact of the measurement process. The object is pushed toward the second hand until the hands get too close to each other. For small objects, the pushing stops before the finger touches the object. This produces always the same sensor data for small objects, which makes it easy to distinguish small from big objects. It further demonstrates the strength of our model-free approach as the exact semantics of the measurement is not required.

The *simple grasp*/*book grasping* behavior is used for small/big objects, respectively. We emphasize that the teaching of novel skills does not necessarily have to follow the typical sequence of *sensing* → *preparation* → *basic behavior*, e.g., in *complex grasping* (no basic behavior) and *shelf alignment* (no sensing). In the *complex grasping* task the basic behavior is the *void* behavior, which causes the robot to coordinate different grasping procedures for small and big objects.

#### 11.2.5. Shelf Placement

The task is to place an object in a shelving bay, which is executed using a DMP. The robot uses the *weigh* sensing behavior to determine whether or not an object is already grasped. The *complex grasp* skill/*void* behavior is used if the object is not grasped/grasped, respectively. Note that training of this skill can result in a local maximum, e.g., by choosing the behaviors *simple grasp* or *book grasp*, in particular if the reward is chosen too high. In case the robot trained the skill with a small object, the robot might learn to use the *simple grasp* behavior to pick up the object and therefore is not able to place bigger objects like books on the shelf. However, this can be unlearned by letting the robot play with a bigger object, which will cause the robot to forget to use the *simple grasp* behavior through punishment and to use the *complex grasp* behavior instead.

#### 11.2.6. Shelf Alignment

The task is to push an object on a shelf toward the wall to make space for more objects. The basic behavior is a DMP moving the hand from the left end of the shelve bay to the right end until a certain force is exceeded. As there is no object in front of the robot, all sensing actions except *no sensing* fail. The sensing behavior with the strongest discrimination score is *no sensing* with only one perceptual state and *shelf placement* as preparatory behavior.

#### 11.2.7. Tower Disassembly

The task is to disassemble a stack of up to three boxes. The basic behavior is the *void* behavior. The perceptual states correspond to the number of boxes in the tower. Reward is given in case the tower is completely disassembled. After training, the sensing behavior used is *poking* to estimate four different states, i.e., height *h* ∈ {0, 1, 2, 3}, as a different height of a tower results in different joint/Cartesian positions. The tower cannot be removed with any single available preparatory behavior and therefore the task is *not solvable* with our original approach. Instead, using the behavior composition mechanism, the robot generates combinations of *simple placement, shelf placement* and *shelf alignment* of the form given by the expression

(24) $$\begin{array}{l} {\text{simple placement}}^{*}\left[\text{void}|\text{shelf placement}|\text{shelf alignment}\right] \end{array}$$

This task can only be solved by using the composite behavior generation method, as for towers of height *h* > 1 several pick-and-place actions are required. The generated behavior compositions of the form given in Equation (24) contain the skills *shelf placement* and *shelf alignment* only at the end of the sequence. The reason is that these skills can only remove a box in a controlled way if only one box is left, i.e., *h* = 1. Higher towers are collapsed because of the *complex grasping* skill, which is used by *shelf placement*. It uses *sliding* to estimate the object's size and therefore pushes the tower around. Moreover, the resulting behavior sequence depends on the individual history of the robot. For example, the sequences (*simple placement, simple placement, simple placement*) and (*shelf placement, simple placement, simple placement*) both yield success for *h* = 3. The autonomy of our approach can also be reduced in such a scenario, as several behaviors destroy the tower and require a human to prepare it again. This requires a human in the playing loop, in particular if the required states cannot be prepared by the robot itself.

### 11.3. Simulated Skill Learning

In order to evaluate the effect of active learning and behavior composition on the convergence behavior, a large number of agents have to be trained (data can be found in Supplementary Material). We achieve this by simulating general skills that use the experience on the success rates of the used sub-behaviors (95%) gathered during the real-world experiments. As real-world experiments show, typically there is one sensing behavior *s* that enables the robot to estimate a useful aspect of the environment. For this sensing behavior, we emulate the environment by adding *J*_changing_ preparatory behaviors *b*_*i*_ that change the environment with

(25) $$\begin{array}{l} e_{j}\overset{b_{i}}{\to }e_{\left(j+i\right)\text{\%}L^{s}} \end{array}$$

where $e_{j},e_{\left(j+i\right)\text{\%}L^{s}}$ are perceptual states of the sensing behavior *s*, and *L*^*s*^ is the number of perceptual states of *s*. For all other sensing behaviors, we assume that the perceptual state cannot be estimated reliably and therefore the outcome of any preparatory behavior is random. For the failure cases, i.e., 5% of the preparatory behavior executions, we simulate a random resulting perceptual state. In order to assess the worst-case convergence behavior, we add *J*_stable_ preparatory behaviors that do not change the perceptual state. This is the worst case, as our active learning/behavior composition system cannot use such behaviors to generate different perceptual states. The total number of preparatory behaviors is *J* = *J*_changing_+*J*_stable_. We choose one perceptual state *e*_0_ as state that will be rewarded, i.e., if a preparatory behavior leads to the state *e*_0_, the skill execution will be rewarded. The success rate is simulated and averaged for *N* = 5, 000 robots for different numbers of preparatory behaviors.

Note that most of the environments of the real-world experiments shown in section 11.2 can be formulated as systems that follow a similar behavior. For example the dominant sensing behavior in the book grasping scenario (c.f. Figure 6B) is the *sliding* behavior. The *rotate 90*° behavior performs a transition between neighboring perceptual states. We therefore emphasize the generality of the simulated experiments.

The number of rollouts required to reach a success rate of at least 90% is given in Table 2 for an increasing number of behaviors *J* and different variants of our method (*N*_no_ext_ ≡ no active learning/no behavior composition ≡ basic approach by Hangl et al. (2016), *N*_active_ ≡ active learning/no behavior composition, *N*_comp_ ≡ no active learning/behavior composition, *N*_full_ ≡ active learning/behavior composition, *N*_trial_error_ ≡ baseline). As baseline we use a trial-and-error policy in which every combination of perceptual states and behaviors is tried out only once, with *N*_trial_error_ = 3 × 4 × *J* + *J* (3 sensing behaviors with 4 states, 1 sensing behavior, i.e., *no sensing* with only one state). In general, our method converges faster than the baseline by reducing the space strongly and ignoring irrelevant parts of the ECM. Further, the baseline method would not yield convergence in a scenario with possible execution failures as each combination is executed only once. The baseline approach also cannot solve the task in the setting with a reduced number of behaviors.

**Table 2:** Number of rollouts required to converge to a success rate of at least 90% for different numbers of additional behaviors *J*_stable_.

**J**	***J*_stable_**	***N*_no_ext_**	***N*_active_**	***N*_comp_**	***N*_full_**	***N*_trial_error_**
4/2	1	37	34	49	46	52
9/7	6	87	67	113	101	117
14/12	11	137	102	177	156	182
19/17	16	189	139	247	215	247
24/22	21	241	176	312	278	312
29/27	26	292	214	390	342	377

*The two numbers in the column J denote the different numbers of behaviors for the settings with and without behavior composition in order to make the two settings equally expressive. The trial and error baseline could not solve the problem in the composite generation setting*.

**Figure 7:**
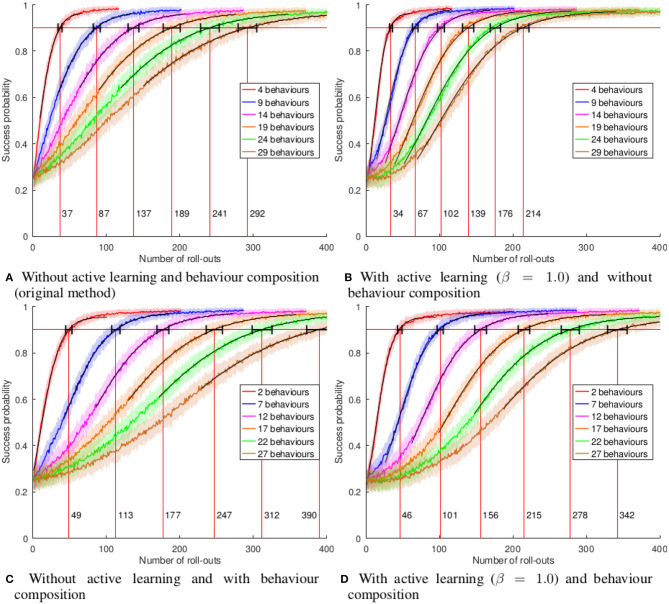
Evolution of the simulated success rates over the number of rollouts for different numbers of preparatory behaviors. For each curve from left to right, five behaviors are added. The red horizontal lines denote an average success rate of 90%. The total number of behaviors is given by *J* = *J*_changing_ + *J*_stable_, where *J*_changing_ denotes the number behaviors that change the perceptual state and *J*_stable_ denotes the number of behaviors that leave the perceptual state unchanged. Without behavior composition we chose *J*_changing_ = 4 so that the target state can be reached from every state. With behavior composition we chose *J*_changing_ = 2 (one behavior to hop to the next state and the *void* behavior) which makes both settings equally expressive. Note that the setting with behavior composition is significantly harder as not all required preparatory behaviors are available to the robot. To robustly estimate the point of convergence, a sigmoid model of the form sig[*c*_1_ − *c*_2_*x*] + *c*_3_ (plotted in black) was fit for the interval [*x*_conv_ − Δ*x, x*_conv_ + Δ*x*] of each curve. *x*_conv_ is the first point for which the average success rate surpasses 0.9 and Δ*x* is 25% of the total length of the curve. **(A)** Without active learning and behavior composition (original method). **(B)** With active learning (β = 1.0) and without behavior composition. **(C)** Without active learning and with behavior composition. **(D)** With active learning (β = 1.0) and behavior composition.

**Figure 8:**
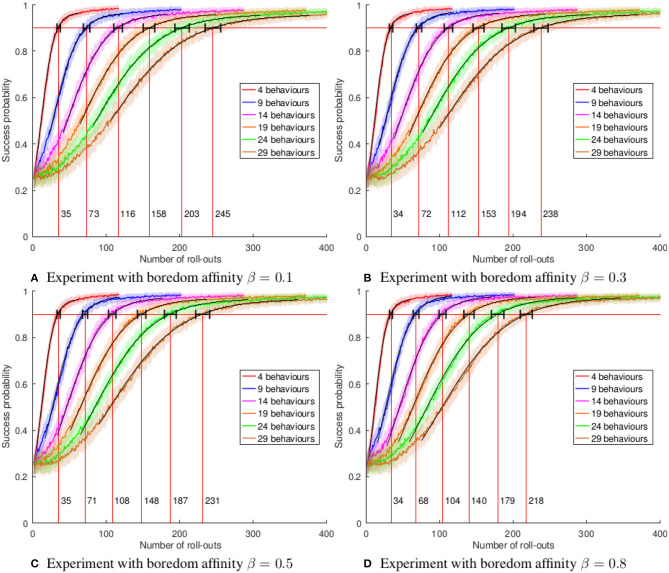
Evolution of the simulated success rates over the number of rollouts for different numbers of preparatory behaviors with different values for the *boredom affinity* β parameter. For each curve from left to right, five behaviors are added. The red horizontal lines denote an average success rate of 90%. The selected parameter values were β ∈ {0.1, 0.3, 0.5, 0.8, 1.0}. The graph for β = 1.0 is shown in Figure 7B. Increase of affinity yields a significant increase of the learning speed proportional to β. **(A)** Experiment with boredom affinity β = 0.1. **(B)** Experiment with boredom affinity β = 0.3. **(C)** Experiment with boredom affinity β = 0.5. **(D)** Experiment with boredom affinity β = 0.8.

**Figure 9:**
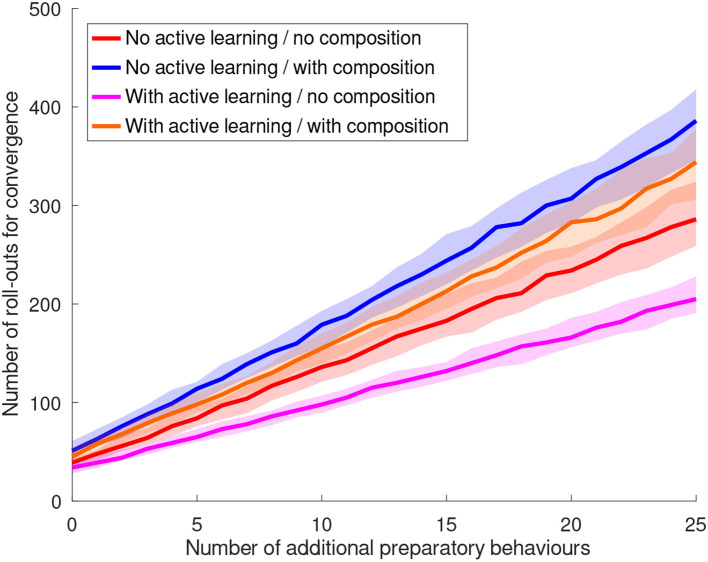
Comparison of the convergence of the success rates between the original method and the extensions. The two curves with behavior composition solve a harder problem in which not all preparatory behaviors are available at the beginning. A linear fit *k*_*i*_*x* + *d*_*i*_ was performed for all these curves (not shown) with the coefficients (*k*_1_, *d*_1_) = (10, 36) (no active learning, no behavior composition), (*k*_2_, *d*_2_) = (7, 31) (with active learning, no behavior composition), (*k*_3_, *d*_3_) = (13, 46) (no active learning, with behavior composition), and (*k*_4_, *d*_4_) = (12, 40) (with active learning, with behavior composition).

The two versions without behavior composition, i.e., without and with active learning, show continuous increase of the success rate in Figures 7A,B. If the robot is bored, situations with a low information gain are rejected. Therefore, the version with active learning is expected to converge faster. Figure 9 shows the number of required rollouts to reach a success rate of 90% for each of the four variants in Figure 7. The number of required rollouts is proportional to the number of available preparatory behaviors. We apply a linear fit and gain an asymptotic speed-up of $sp=1-\underset{x\to \infty }{\lim}\frac{k_{1}x+d_{1}}{k_{2}x+d_{2}}=1-\frac{k_{1}}{k_{2}}\approx 30$% for the variant with active learning with the most optimal boredom affinity β = 1.0 compared to the variant without extension. By using more conservative values for the boredom affinity, the speed-up decreases as shown in Figure 8. Statistical analysis was performed to demonstrate the significance of the effect (c.f. Table 3).

**Table 3:** Pairwise *t*-test results for the convergences derived from the experiments shown in Figure 8.

	**β_1_ = 0.1**	**β_2_ = 0.3**	**β_3_ = 0.5**	**β_4_ = 0.8**	**β_5_ = 1.0**
β_1_ = 0.1	0.00	0.69	0.85	0.96	1.00
β_2_ = 0.3	0.69	0.00	0.54	0.92	0.96
β_3_ = 0.5	0.85	0.54	0.00	0.65	0.96
β_4_ = 0.8	0.96	0.92	0.65	0.00	0.46
β_5_ = 1.0	1.00	0.96	0.96	0.46	0.00

*For each pair of convergences matching by number of behaviors J ∈ [2, 29] between experiments with different values β, a t-test was performed. This table shows the fraction of points for which the improvement was statistically significant (significance level of 0.05). The analysis shows that the improvement becomes more significant the higher the difference Δβ = β_j_ − β_i_ is*.

Active learning can also be combined together with behavior composition, in which the speed-up reaches *sp* = 8% (c.f. Figures 7C,D). As both mechanisms, active learning and behavior composition, exploit the same model, the reduced speed-up can be expected. As soon as the environment model is mature enough to plan trajectories through the perceptual state space, it can likely be used to generate behaviors that solve the task in given states.

**Figure 10:**
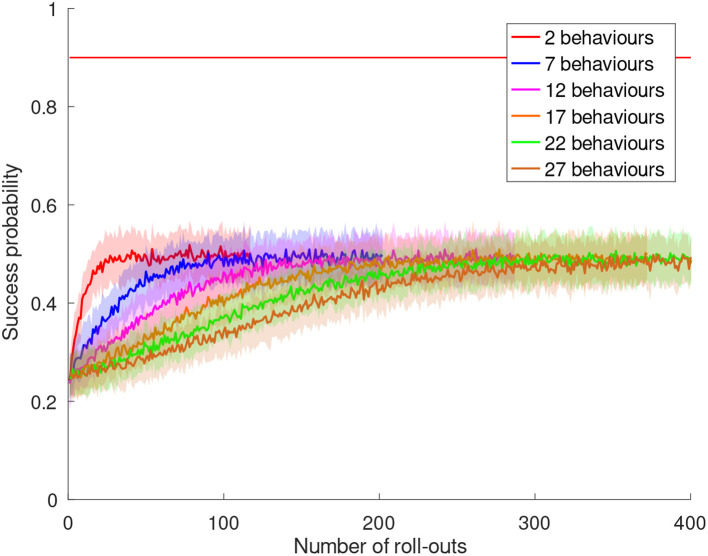
Success rates without active learning and without behavior composition in a setting that requires behavior composition, i.e., not all preparatory behaviors required to solve the task are available. In this case, the *void* behavior and the behavior to transition to the neighboring perceptual state are available. This enables the agent to solve the task for two out of four perceptual states.

In the scenario with activated behavior composition the convergence behavior is different (c.f. Figures 7C,D). It is important to note that in this scenario not all preparatory behaviors required to solve the task were available initially, as the robot is only provided with a behavior to state at the current state (*void* behavior) and a behavior that allows it to jump from the current perceptual state to the next neighbor state. The success rate exhibits a slow start followed by a fast increase and a slow convergence toward 100%. The slow start is due to the perceptual states that would require the missing behaviors. At this point the robot cannot generate these behaviors using behavior composition due to initially untrained environment models. This causes the success rate to reach a preliminary plateau at around 35%, this is in particular visible for higher numbers of *J*. After this initial burn-in phase, the environment model becomes more mature and behavior proposals are created which causes a strong increase of the success rate. With the original approach this tasks is not solvable for all situations as it is shown in Figure 10.

## 12. Conclusion

We introduced a novel way of combining model-free and model-based reinforcement learning methods for autonomous skill acquisition. Our method acquires novel skills that work for only a narrow range of situations acquired from a human teacher, e.g., by demonstration. Previously-trained behaviors are used in a model-free RL setting in order to prepare these situations from other possibly occurring ones. This enables the robot to extend the domain of applicability of the novel skill by playing with the object. We extended the model-free approach by learning an environment model as a byproduct of playing. We demonstrated that the environment model can be used to improve the model-free playing in two scenarios, i.e., active learning and composite behavior generation. In the active learning setting the robot has the choice of rejecting present situations if they are already well-known. It uses the environment model to autonomously prepare more interesting situations. Further, the environment model can be used to propose novel preparatory behaviors by concatenation of known behaviors. This allows the agent to try out complex behavior sequences while still preserving the model-free nature of the original approach.

We evaluated our approach on a KUKA robot by solving complex manipulation tasks, e.g., complex pick-and-place operations, involving non-trivial manipulation, or tower-disassembly. We observed success statistics of the involved components and simulated the convergence behavior in increasingly complex domains, i.e., a growing number of preparatory behaviors. We found that by active learning the number of required rollouts can be reduced by ~30%. We have shown that composite behavior generation enables the robot to solve tasks that would not have been solvable otherwise, e.g., complex book grasping with a reduced number of preparatory behaviors or tower disassembly.

The work presented in this paper bridges the gap from plain concatenation of pre-trained behaviors behaviors to simple goal-directed planning. This can be seen as early developmental stages of a robot. We believe that a lifelong learning agent has to go through different stages of development with an increasing complexity of knowledge and improving reasoning abilities. This raises the question of how the transition to strong high-level planning systems could look like.

Our experiments show that the learning time is proportional to the number of used preparatory behaviors. This makes it efficient to learn an initial (and potentially strong) set of skills, but hard to add more skills when there is a large set of skills available already. Training more sophisticated models could help to overcome this problem. Further, in the current system, the composite behavior generation only allows behavior compositions resulting from plans within the same environment model, i.e., using only perceptual states of the same sensing behavior. The expressive power of our method could be greatly increased by allowing plans through perceptual states of different sensing behaviors. This could also involve multiple sensing behaviors at the same time including passive sensing, such as vision.

## Data Availability Statement

The raw data supporting the conclusions of this article will be made available by the authors, without undue reservation, to any qualified researcher. Further, all generated data can be re-generated by using the publicly available source code of the *kukadu* framework^1^.

## Author Contributions

SH was responsible for the overall design, implementation, and evaluation of the presented method. VD and HB contributed with ideas to design and improve the methods and support on the application of projective simulation to robotics and provided the overall guidance and supervision. JP contributed with ideas to design and improve the methods and their positioning inside the current body of robotics research and provided the overall guidance and supervision.

### Conflict of Interest

The authors declare that the research was conducted in the absence of any commercial or financial relationships that could be construed as a potential conflict of interest.

## Footnotes

^1^https://github.com/shangl/kukadu

^2^https://www.youtube.com/watch?v=8G-vqAWtRCE

^3^https://iis.uibk.ac.at/public/shangl/iros2016/iros.mpg
